# Does Use of Acupuncture Reduce the Risk of Type 2 Diabetes Mellitus in Patients With Rheumatoid Arthritis? Evidence From a Universal Coverage Health Care System

**DOI:** 10.3389/fmed.2021.752556

**Published:** 2021-10-21

**Authors:** Wei-Jen Chen, Hanoch Livneh, Chi-Hsien Chen, Hui-Ju Huang, Wen-Jiun Liu, Ming-Chi Lu, Tzung-Yi Tsai

**Affiliations:** ^1^Department of Chinese Medicine, Dalin Tzuchi Hospital, The Buddhist Tzuchi Medical Foundation, Chiayi, Taiwan; ^2^Graduate Institute of Sports Science, National Taiwan Sport University, Taoyuan, Taiwan; ^3^School of Post-baccalaureate Chinese Medicine, Tzu Chi University, Hualien, Taiwan; ^4^Center of Sports Medicine, Dalin Tzuchi Hospital, The Buddhist Tzuchi Medical Foundation, Chiayi, Taiwan; ^5^Rehabilitation Counseling Program, Portland State University, Portland, OR, United States; ^6^Division of Cardiology, Department of Internal Medicine, Dalin Tzuchi Hospital, The Buddhist Tzuchi Medical Foundation, Chiayi, Taiwan; ^7^Department of Nursing, Dalin Tzuchi Hospital, The Buddhist Tzuchi Medical Foundation, Chiayi, Taiwan; ^8^Division of Allergy, Immunology and Rheumatology, Dalin Tzuchi Hospital, The Buddhist Tzuchi Medical Foundation, Chiayi, Taiwan; ^9^School of Medicine, Tzu Chi University, Hualien, Taiwan; ^10^Department of Environmental and Occupational Health, College of Medicine, National Cheng Kung University, Tainan, Taiwan; ^11^Department of Nursing, Tzu Chi University of Science and Technology, Hualien, Taiwan; ^12^Department of Medical Research, Dalin Tzuchi Hospital, The Buddhist Tzuchi Medical Foundation, Chiayi, Taiwan

**Keywords:** rheumatoid arthritis, type 2 diabetes mellitus, acupuncture, risk, cohort study

## Abstract

**Objectives:** Although acupuncture is often advocated for patients with rheumatoid arthritis (RA), its efficacy for type 2 diabetes mellitus (T2DM), a common metabolic disease among RA cohorts, has not yet been established. This retrospective cohort study aimed to determine the association between acupuncture use and the development of T2DM among them.

**Methods:** Data were collected from 1999 through 2008 for individuals aged 20–70 years in the nationwide insurance database of Taiwan. From them, we extracted 4,941 subjects within newly diagnosed RA and being T2DM free at baseline. A total of 2,237 patients had ever received acupuncture, and 2,704 patients without receiving acupuncture were designated as a control group. All of them were followed to the end of 2013 to identify T2DM incidence. The Cox proportional hazards regression model was utilized to obtain the adjusted hazard ratio (HR) for acupuncture use.

**Results:** Compared with the RA subjects without use of acupuncture, the incidence of T2DM was lower for those who received acupuncture, with the incidence rates of 24.50 and 18.00 per 1,000 person-years (PYs), respectively. After adjusting for potential confounders, use of acupuncture was significantly related to the lower T2DM risk, with the adjusted HR of 0.73 [95% confidence interval (CI) 0.65–0.86]. Those who used acupuncture for more than five sessions had the greatest benefit in lowering the susceptibility to T2DM.

**Conclusion:** Adding acupuncture into conventional treatment for RA was found to be related to lower risk of T2DM among RA patients. Further clinical and mechanistic studies are warranted.

## Highlights

- Acupuncture, a key component of traditional Chinese medicine, are being increased consideration in clinical practice against the onset of inflammatory diseases.
- As of now, no study was conducted to determine the long-term effect of acupuncture against the T2DM development among rheumatoid arthritis (RA) subjects, who are at higher risk of developing T2DM due to augmented systemic inflammation.
- Among the recruited RA subjects, 2,237 (45.3%) ever received acupuncture and had a lower risk of T2DM than those without using acupuncture by 27%.
- Those who used acupuncture treatments for more than 5 sessions had the greatest benefit in lowering the susceptibility to T2DM.
- Results issued from larger sample sizes are be given more credit while determining the effect of acupuncture on the glycemic control, particularly among RA subjects.

## Introduction

Rheumatoid arthritis (RA) is a chronic disease that affects about 1% of the population worldwide, with many patients ultimately developing progressive functional limitations and physical disability (1). These phenomena, together with accompanying side effects, can affect the RA patient's work and productivity. It was estimated that approximately one-third of affected individuals are unable to work within 3 years of the onset of RA (2), thus causing enormous socioeconomic burden. A recent study by Birnbaum and co-workers noted that the total annual cost of RA in the US was around $19.3 billion, and adding the intangible costs of quality-of-life deterioration ($10.3 billion) and premature mortality ($9.6 billion), the total annual societal costs of RA (direct, indirect, and intangible) would rise to nearly $40 billion (3).

It is well-known that RA may trigger a number of crippling comorbidities in addition to the enormous economic burdens. Recent evidence has indicated a positive association between the autoimmune rheumatic diseases and cardio-metabolic comorbidities, especially T2DM, commonly seen with aging (4). One meta-analysis of 19 studies found that persons with a history of RA were nearly 30% more likely to have T2DM than those without RA (5). To make matters worse, the coexistence of RA and diabetes would insidiously increase subsequent mortality risk (4). Several scholars concluded that the link between these two diseases might be related to plasma inflammation (6). Additionally, the known risk of cardiovascular comorbidity for RA patients may be implicated in their susceptibility to T2DM.

Over the past few years, acupuncture has been employed as a treatment modality for numerous diseases (7, 8). For example, a meta-analysis of 43 studies containing 1,722 RA patients found that adding acupuncture to routine medical care could bring clinically relevant benefits, like improved physical function and quality of life (9). Notably, based on earlier research, the regulation of innate inflammatory responses from acupuncture treatment was proposed to achieve favorable therapeutic outcomes among the subjects with inflammatory conditions by modulating inflammation signaling pathway (10–12). Given that there is growing evidence indicating the abnormal inflammatory responses may be involved in the link between RA and diabetes (13), the application of acupuncture might be considered while instituting the appropriate care regimen for preventing or delaying the incidence of diabetes among RA patients.

While a substantial number of studies on acupuncture's effects have been conducted, most have been performed in Western countries. Moreover, these studies were limited to data derived from self-report questionnaires and chart reviews, and were based on small sample sizes (11, 14). Notwithstanding these studies, the association between acupuncture use and T2DM risk among RA patients is still unknown. To derive a more precise estimation of acupuncture's effect on subsequent development of T2DM among individuals with RA, we conducted this cohort study using a nationwide claims data from Taiwan.

## Methods

### Database

Data used in this retrospective cohort study were retrieved from the national representative sample of the Longitudinal Health Insurance Database (LHID), which comprises a sub-dataset of the National Health Insurance (NHI) program of Taiwan, made up of one million randomly sampled people with over 10 years of follow-up. It has also been reported that these 1 million insured individuals are of similar age and sex distribution to the general population of Taiwan because these data were obtained by the Bureau of NHI based on a multistage stratified systematic sampling method (15). This database compiles (i) demographic information of enrollees; (ii) health insurance claims data; (iii) diagnostic codes; (iv) contracted pharmacies; and (v) medical examination information from the NHI program. Researchers using the LHID are required to follow closely the Taiwan Personal Information Protection Act. Not only being conducted in accordance with the Helsinki Declaration, this study was also approved by the local institutional review board and ethics committee of Buddhist Dalin Tzu Chi Hospital (No. B10803015-1).

### Participants

**Figure 1:**
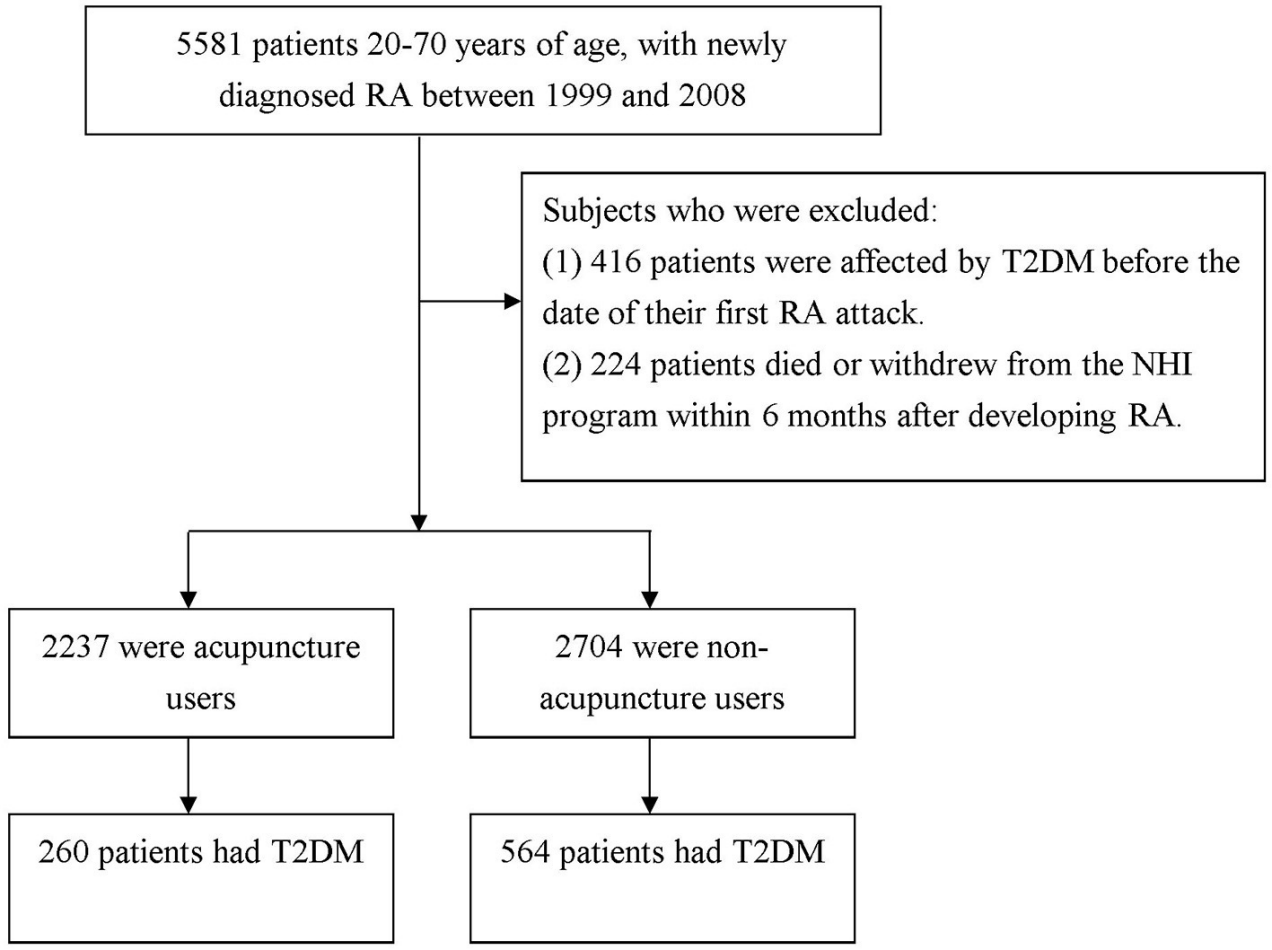
Flowchart of selection and follow-up of study subjects.

In this exploration, the diagnostic codes were based on the International Classification of Disease, Ninth Revision, Clinical Modification (ICD-9-CM). As shown in Figure 1, study participants were included if they were 20–70 years old, and had a new diagnosis of RA (ICD-9-CM 714.0) in one hospitalization or ≥3 outpatient visits between 1999 and 2008 (*n* = 5,581). These patients were further linked to the catastrophic illness registry to ensure the validity of diagnosis. The catastrophic illness certificate is granted based on formal diagnoses issued by physicians, such as schizophrenia, mood disorders, autoimmune disorders, or cancer. The date when RA patients gained the approval for catastrophic illness registration was considered as the index date. Among them, a total of 416 patients, diagnosed with T2DM before the date of T2DM onset were deleted. The diagnostic algorithm to determine T2DM requires at least 3 outpatient visits, or at least one inpatient claim for the ICD-9-CM codes of 250X0 or 250X2. Also excluded were individuals with missing data and those who were not followed for at least 6 months after RA onset (*n* = 224). Following this step, the remaining subjects were linked to the ambulatory care visit claims to identify if they ever received acupuncture treatment after RA onset.

In this study, acupuncture treatment was identified when acupuncturists used commercially available, single-use, sterile, disposable stainless-steel needles. According to the program's protocol, when 6 acupuncture treatments are consecutively delivered within 1 month to a patient, they are defined as a package of acupuncture (16). Those receiving at least 1 package of acupuncture due to an RA diagnosis were identified as “acupuncture users,” while the remainder were regarded as “non-acupuncture users.” Patients who were not followed for a full 6 months after discharge were excluded because the related treatment program for RA was considered most effective within the first 6 months after RA onset (17). In addition, considering that immortal time bias may induce spurious results in the treatment group (18), the index date of follow-up period for those who were classified as non-acupuncture users was assigned to the date of the first RA made, whereas the index date of follow-up period for RA patients who used acupuncture was assigned to the first date of the initiation of acupuncture treatment. Follow-up person-years (PYs) for both groups were calculated from the index date to the earliest of one of the following: the onset of T2DM (ICD-9-CM codes: 250X0 or 250X2), the date of withdrawal from the insurance plan, or the date of December 31, 2013.

### Definitions of Covariates

Covariates assessed included baseline age, sex, monthly income, urbanization level of enrollee's residential area, former comorbidities, and medication usage. Regarding monthly income, we used the premium category as a proxy to divide participants into four groups: ≤ New Taiwan Dollar (NTD) 17,800, NTD 17,881-43,900, and ≥ NTD $43,901. As to the urbanization level, it was calculated according to a published scheme that classified 359 communities in Taiwan into seven stratums, with a higher level indicating a higher degree of urbanization. The classification scheme included the population density, proportion of persons with a college-level education or higher, proportion of elderly residents, proportion of agricultural workers, and number of physicians per 100,000 population. In this work, the urbanization degree was grouped into three strata: urban (levels 1–2), suburban (levels 3–4), and rural (levels 5–7). Monthly incomes were stratified into 3 levels: (1) ≤ New Taiwan Dollar (NTD) 17,880, (2) NTD 17,881–43,900, and (3) ≥ NTD 43,901. Urbanization levels were divided into 3 strata, namely, urban, or levels 1–2; suburban, or levels 3–4; and rural, levels 5–7. Level 1 refers to the “most-urbanized” and level 7 refers to the “least-urbanized” communities (19). Baseline comorbidities for each subject were determined by individual medical records in the year preceding cohort entry, all of which were assessed by the established Charlson-Deyo comorbidity index (CCI) (20). The CCI score was a composite of 17 chronic diseases, each with a score of 1-to-6 points. The sum of these scores was regarded as a continuous variable for the burden of comorbidities, with higher scores indicating a more severe impact of these comorbidities. To avoid duplicate counting and possible over-adjustment in the regression model, RA and T2DM were excluded from the CCI score. Additionally, medication use was stratified into two categories based on whether, or not, the subject ever used disease-modifying anti-rheumatic drugs, such as methotrexate, sulfasalazine, ciclosporin, hydroxychloroquine, penicillamine and leflunomide, for more than 6 months after the index date.

### Statistical Analysis

Categorical variables are reported as frequency and/or percentage, and the continuous variables are presented as mean with standard deviation (SD). In step one of the analysis, the Chi-square test and *t*-test were used to examine the differences in demographic variables and comorbidities between those receiving and not receiving acupuncture. The incidence rates of T2DM were presented with the number of cases per 1,000 PYs. The difference in the cumulative incidence of T2DM between these two groups was calculated with the Kaplan-Meier method and tested using the log-rank test. Cox proportional hazards regression analysis was further applied to compute the hazard ratio (HR), with 95% confidence intervals (CI) of risk of events in association with acupuncture use. Stratified analysis by age and gender was examined as well. The proportional-hazards assumption was tested by plotting log [–log (survival probability)] vs. log (survival time) curves. All analyses were conducted using SAS version 9.3 (SAS Institute Inc., Cary, NC, USA), at a *P* < 0.05 statistical significance level.

Additionally, two sensitivity analyses were done to meticulously evaluate if the association found was robust. First, given on the unavailability of RA severity in this investigation, we added the prescription of biological agents, used for 6 months or longer, as a surrogate variable for RA severity into the regression model. These agents contained adalimumab, etanercept, infliximab, rituximab and tocilizumab. Second, to make both samples comparable, we utilized the propensity score with a 1:1 matching method to balance the characteristics between the acupuncture and non- acupuncture groups. The multivariable logistic regression model was utilized to estimate PS value, a probability index, ranging between 0 and 1, and derived from the influence of all observed characteristics (listed at Table 1). Patients were matched by propensity score using one-to-one nearest neighbor matching within 0.2 caliper distance, in which pairs of user and nonuser groups were formed, such that matched subjects have similar values of the propensity scores.

**Table 1:** Demographic variables and comorbidities of RA subjects who received and did not receive acupuncture use.

**Variable**	**Non-acupuncture**	**Acupuncture**	***P***
	**users (%)**	**users (%)**	
	**(*n* = 2,704)**	**(*n* = 2,237)**	
Age (year)			<0.001
≤ 50	1,241 (45.9)	1,211 (54.1)	
>50	1,463 (54.1)	1,026 (45.9)	
Mean (Standard Deviation, SD)	50.8 (12.1)	48.8 (11.5)	<0.001
Gender			<0.001
Female	1,777 (65.7)	1,658 (74.1)	
Male	927 (34.2)	579 (25.9)	
Monthly income			0.26
Low	1,082 (55.2)	944 (42.2)	
Median	1,491 (55.1)	1,195 (53.4)	
High	131 (4.8)	98 (4.4)	
Residential area			0.001
Urban	1,493 (55.2)	1,307 (58.4)	
Suburban	418 (15.5)	374 (16.7)	
Rural	793 (29.3)	556 (24.9)	
Medication use			0.17
Yes	2,255 (83.4)	1,832 (81.9)	
No	449 (16.6)	405 (18.1)	
CCI	4.09 (6.14)	3.81 (5.11)	0.09

## Results

We identified 4,941 Taiwanese patients in the LHID who had been diagnosed with RA between 1998 and 2007. The data from the acupuncture and non-acupuncture cohorts yielded information for 2,237 and 2,704 subjects, respectively. Details of the demographic variables and comorbidities among them are shown in Table 1. Compared to non-acupuncture users, acupuncture users were more likely to be younger, female, and residing in an urban area.

**Figure 2:**
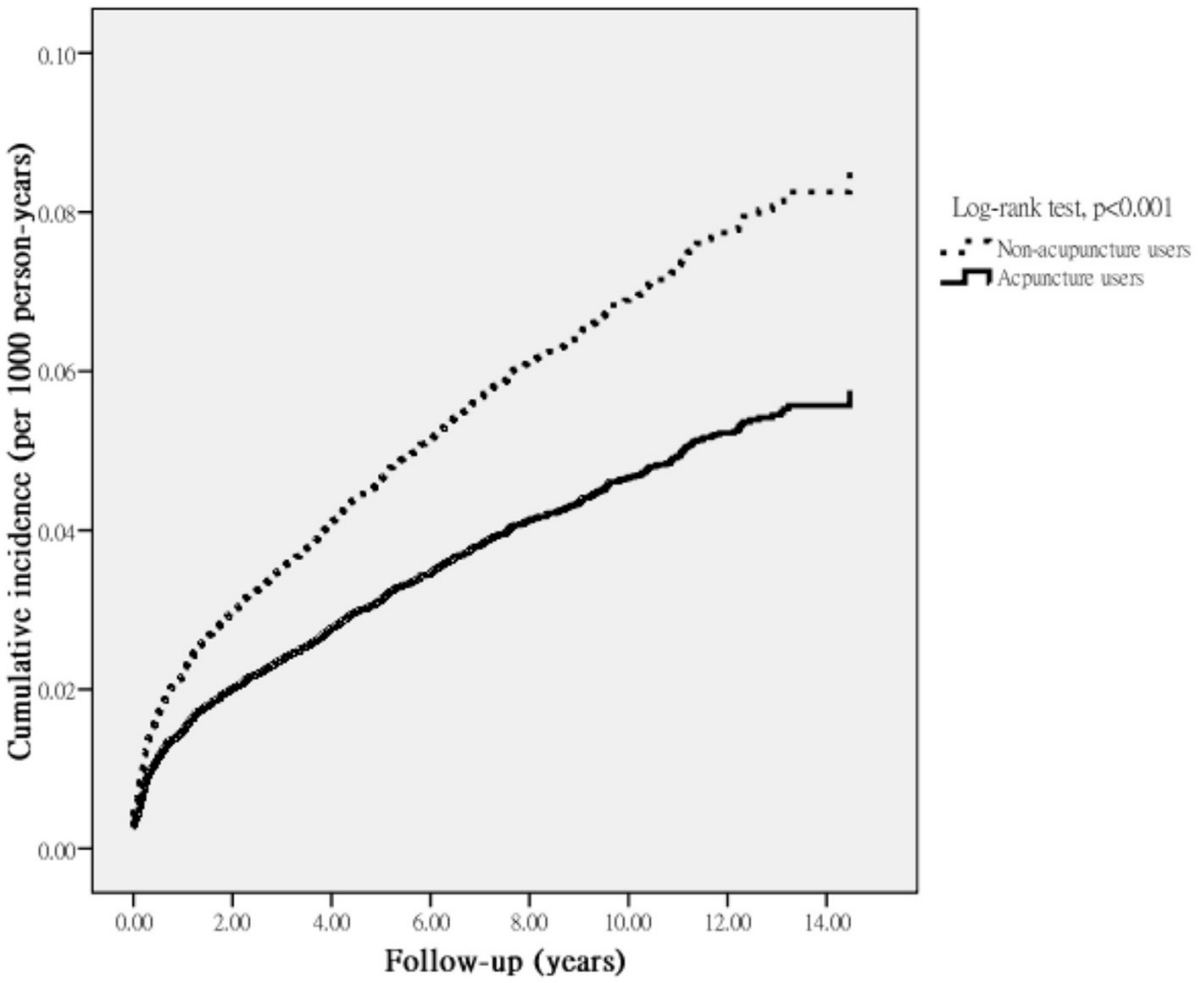
Cumulative incidence of T2DM among RA patients who received or did not receive acupuncture.

During the follow-up period, a total of 824 first episodes of T2DM occurred among all eligible subjects, including 564 episodes among non-acupuncture users and 260 among acupuncture users, during the follow-up periods of 23,021.97 and 14,442.43 PYs, respectively. Compared to RA patients who did not use acupuncture, those using acupuncture exhibited a reduced incidence of T2DM (18.00 vs. 24.50 incidents per 1,000 PYs). After adjusting for gender, age, monthly income, urbanization, and comorbidities, those who used acupuncture had a lower risk of T2DM (adjusted HR 0.73; 95% CI: 0.65–0.86) (Table 2). Results of the Kaplan-Meier survival curve and log-rank tests also supported a statistically significant difference in the survival rate free from T2DM between two groups within the study period, showing those receiving acupuncture substantially exhibited a significantly lower incidence rate of T2DM (*P* < 0.001) (Figure 2).

**Table 2:** Overall age- and sex-specific incidence and adjusted HR of T2DM in relation to acupuncture treatment among RA patients.

**Variables**	**Non-acupuncture users**			**Acupuncture users**			**Crude HR (95% CI)**	**Adjusted HR (95% CI)**
	**T2DM**	**PYs**	**Incidence**	**T2DM**	**PYs**	**Incidence**		
Female age (year)								
< =50	94	7,276.74	12.92	72	5,995.34	12.01	0.92 (0.67–1.23)	0.90^*^ (0.65–1.22)
>50	270	7,729.37	34.93	124	4,738.20	26.17	0.73 (0.57–0.86)	0.73^*^ (0.59–0.98)
All	364	15,006.11	24.26	196	10,733.54	18.26	0.72 (0.60–0.85)	0.77^**^ (0.66–0.91)
Male age (year)								
< =50	124	4,076.78	30.42	47	1,655.11	28.40	0.91 (0.63–1.24)	0.89^*^ (0.62–1.23)
>50	76	3,939.08	19.29	17	2,053.78	8.28	0.45 (0.25–0.73)	0.45^*^ (0.26–0.75)
All	200	8,015.86	24.95	64	3,708.89	17.26	0.69 (0.50–0.88)	0.71^**^ (0.53–0.92)
Overall	564	23,021.97	24.50	260	14,442.43	18.00	0.71 (0.61–0.82)	0.73^***^ (0.65–0.86)
PS matching	415	18,699.06	22.19	253	14,043.35	18.01	0.77 (0.66–0.90)	0.78^***^ (0.68–0.91)

*Incidence rate is per 1,000 PYs*.

* *Model adjusted for urbanization level, monthly income, medication usage and CCI*.

** *Model adjusted for age, urbanization level, monthly income, medication usage and CCI*.

*** *Model adjusted for age, sex, urbanization level, monthly income, medication usage and CCI*.

*PS matching, propensity score matching*.

Since a reliable index of RA severity was unavailable from the LHID, and failure to adjust for this factor may have affected the findings, we carried out a sensitivity analysis to verify the association between acupuncture use and subsequent risk of T2DM. After utilizing the prescription of biological agents as a surrogate for RA severity, and dividing all enrollees by whether they received biological agents for 6 months after the index date, we found that the proportion of use of biological agents was 55.7% (1,246/2,237) in the acupuncture users and 52.8% (1,428/2,704) in the non-acupuncture users, respectively, and acupuncture significantly and subsequently reduced the risk of T2DM (adjusted HR 0.77; 95% CI: 0.64–0.89). Additionally, the second sensitivity analysis with propensity score matching indicated that 11.6% (253/2,175) acupuncture users and 19.1% (415/2,175) non-acupuncture users developed T2DM. Overall, the acupuncture group was associated with a reduced risk of T2DM, per the adjusted HR of 0.78 (95% CI: 0.68–0.91) (Table 2).

Table 2 showed the results stratified by age and gender. Both female and male RA patients who were receiving acupuncture treatment had a significantly decreased risk of T2DM, with the adjusted HR of 0.77 (95% CI: 0.66–0.93) and 0.71 (95% CI: 0.53–0.92), respectively. In this subgroup analysis, acupuncture use appeared to be more beneficial for males 50 years of age or older (adjusted HR 0.45; 95% CI: 0.26–0.75). On the whole, Table 3 indicated that a strong dose-response effect of post-RA acupuncture, in which the acupuncture users with high intensity had the lower risk of T2DM.

**Table 3:** Risk of T2DM in association with number of packages of acupuncture treatment.

**Number of**	**Number**	**T2DM**	**PYs**	**Incidence**	Adjusted HR^**^
**packages**					**(95% CI)**
0	2,704	564	23,021.97	24.50	1
1	1,536	189	8,930.39	21.16	0.89 (0.76–1.06)
2	271	26	1,671.63	15.55	0.66 (0.45–0.98)
3	216	24	2,005.92	11.96	0.54 (0.30–0.89)
4	108	11	931.20	11.81	0.51 (0.37–0.76)
>=5	106	10	903.28	11.07	0.48 (0.25–0.79)

*Incidence rate is per 1,000 PYs*.

** *Model adjusted for age, sex, urbanization level, monthly income, medication usage and CCI*.

## Discussion

Since diabetes is one of the most commonly observable predictors of cardiovascular disease, which is the primary cause of increased mortality in RA patients, it is valuable to investigate the effect of acupuncture on risk of incident T2DM in RA patients, using a large scale follow-up cohort study.

Over the 15-year study period, we found a nearly 30% lower risk of T2DM among those receiving acupuncture than those not receiving acupuncture. We also noted that use of more than 5 packages of acupuncture decreased the risk of developing T2DM even further, to 52%. Examination of the dose-response relationship further clarified the nature of acupuncture use and the subsequent risk of T2DM. While the related findings on the long-term impact of acupuncture on the prevention of T2DM onset among RA individuals are scarce, this positive therapeutic effect adds to a growing body of literature on the clinical efficacy of acupuncture use among them (9, 11).

We argue that several reasons may be considered to explain the reason that use of acupuncture may influence the reduction of T2DM among RA patients. First, the presence of balance between T-cell subsets Th1 and Th2 cell-derived pro-inflammatory cytokines after acupuncture administration may account for the positive impact of acupuncture. Several animal experiments and human studies reported that the use of acupuncture significantly reduced levels of pro-inflammatory cytokines, such as interleukin (IL)-1β, IL-6, and tumor necrosis factor-α (TNF-α) (21, 22). The possible link between acupuncture use and lower inflammatory parameters may occur through the mediation of the immune system-to-brain pathway (10). Earlier studies showed that acupuncture was capable of modulating the function of the hypothalamic-pituitary-adrenal axis and the peripheral release of cortisol, along with the balance between the sympathetic and parasympathetic branches' activities (23, 24). In addition, the mechanisms by which acupuncture exerts marked anti-inflammatory effects may be related to the inhibition of nuclear factor kappa beta and mitogen-activated protein kinase (12). These physiologic responses were known to mediate the activity of immune cells and levels of inflammatory cytokines in the serum, such as C-reactive protein, IL-6 or TNF-α (25). All of these have been proven to act as a stimulus for the development of insulin resistance (26). Second, an increasing number of studies applying acupuncture to the treatment of diabetes indicated that the clinical benefits may attribute to the control of blood glucose levels, body weight loss, and improvement of insulin resistance. One recent meta-analysis of seven randomized controlled trials revealed that integration of acupuncture into conventional medicine was indeed beneficial in reducing levels of fasting blood glucose (*P* < 0.001), postprandial blood glucose (*P* = 0.0005) and glycated hemoglobin (*P* = 0.004) (27), which in turn decrease the subsequent T2DM risk.

Additionally, the findings of present study revealed that, regardless of gender, only elderly patients benefited the most from acupuncture treatments in preventing the risk of T2DM. Several reasons may account for this finding. First, since the risk of developing T2DM increases with age, the small sample size of T2DM occurrences among younger individuals may have led to the failure to observe any link between acupuncture use and subsequent risk of T2DM. Second, the quick access to, and maintenance of, social network resources in younger subjects may be another reason for this finding (28). Younger RA patients may adapt more easily to the burden incurred by the irreversible nature of RA progression, thereby lessening or moderating the effect of acupuncture. Third, younger females benefitted less from acupuncture, and this finding could be related to inherent estrogen levels. Estrogen has been found to suppress the production of inflammatory mediators, such as IL-1, IL-6 and TNF-α (29), and these parameters are reported to play a decisive role in the pathogenesis of diabetes mellitus (26).

While our study is the first to investigate the association of acupuncture use with the subsequent risk of T2DM, among RA patients, there are important limitations to consider. First, our observations were obtained through a retrospective cohort research design, based on ICD-9-CM diagnostic codes. Thus, relevant cases may have been misclassified. To minimize this bias, we enrolled only persons with new-onset RA or T2DM, and only after the patients had at least three outpatient visits, reporting consistent diagnoses or at least one inpatient admission. It should also be noted that the NHI of Taiwan randomly reviews the charts and audits medical charges to verify the accuracy of claims (15). Additionally, the coding approach and data availability are similar to the two groups, and any misclassification bias, therefore, is likely to have been non-differential, thus possibly diluting the overall HR observed toward the null value and providing a conservative estimate of risk. Second, because a reliable index of RA severity was unavailable from the database used, and failure to control for this factor may have affected the findings, the corresponding sensitivity analysis was then conducted to confirm whether the use of acupuncture could indeed reduce the subsequent risk of T2DM among them. After using the prescription of biological agents as a surrogate for RA severity, the findings of the reanalysis depicted that RA severity did not appreciably impact the relationships reported herein. Third, the LHID lacks information on family history of diseases, lifestyle, body weight, and laboratory data that may potentially prejudice the present findings. Thus, we could not include these variables and adjust them in the multivariate analysis. Nevertheless, in the sensitivity analysis, we added the CCI score into the propensity analysis to arrive at a comparability of both cohorts, and also included the CCI score in the multivariable and stratified analyses. The sensitivity analysis lent further support to the earlier findings that disease severity did not prejudice the current findings. Fourth, although our study revealed a substantial beneficial effect of acupuncture use on reduction of T2DM risk among RA patients, it must be recognized that participants were not initially randomly categorized into users and nonusers. Therefore, caution should be exerted when interpreting the findings. Nevertheless, we did reanalyze the data by applying the propensity score matching approach to reduce the imbalance between two selected groups. For each RA patient who received acupuncture, one control patient was selected via the propensity score, which was the conditional probability of receiving the treatment given the pre-treatment variables. These revised findings still demonstrated that use of acupuncture was related to the reduction risk of T2DM among RA patients after the propensity-matched analysis. Despite this, a randomized controlled trial, encompassing additional countries, is recommended to corroborate the present findings, as well as to uncover the mechanisms that underlie their successful application. These limitations notwithstanding, this study also possessed several strengths. One strength was that the available data were obtained from the NHI database, which is a government-run, single-payer HNI program covering more than 99% of insured persons and healthcare institutes throughout Taiwan. This ensured that the present study was representative of the general population with minimal selection bias. Additionally, the large sample size allowed us to achieve an adequate statistical power for psychometrically sound analysis, especially given the relatively low incidence of RA in the population.

## Conclusion

In conclusion, this study explored if patients with RA who receive acupuncture could experience a reduced risk of T2DM. Although findings indicated the beneficial effect of acupuncture against onset of T2DM event, future prospective randomized trials that overcome the limitations of this study are needed to provide more conclusive evidence of the association suggested herein.

## Data Availability Statement

The data supporting the conclusion of this study are available from the authors, but the raw data (NHIRD) need to be obtained from the National Health Research Institute of Taiwan through an application process upon approval.

## Ethics Statement

The studies involving human participants were reviewed and approved by the Local Institutional Review Board and Ethics Committee of Buddhist Dalin Tzu Chi Hospital. Written informed consent for participation was not required for this study in accordance with the national legislation and the institutional requirements.

## Author Contributions

W-JC, C-HC, M-CL, and T-YT were involved in the study design and drafted the manuscript. HL, H-JH, and T-YT contributed to data analysis and revised the manuscript. C-HC, M-CL, W-JL, H-JH, M-CL, and T-YT contributed to the interpretation of data and provided comments on the final draft of the manuscript. M-CL provided administrative support and comments on the manuscript drafts. T-YT, HL, and W-JC were responsible for the study conception, design, data analysis, and drafting. All authors read and approved the final manuscript.

## Funding

This research was supported by Dalin Tzuchi Hospital (Grant Numbers DTCRD 109-I-28 and DTCRD 110-I-22).

## Conflict of Interest

The authors declare that the research was conducted in the absence of any commercial or financial relationships that could be construed as a potential conflict of interest.

## Publisher's Note

All claims expressed in this article are solely those of the authors and do not necessarily represent those of their affiliated organizations, or those of the publisher, the editors and the reviewers. Any product that may be evaluated in this article, or claim that may be made by its manufacturer, is not guaranteed or endorsed by the publisher.
